# Supplementary material for: Spyglass is a framework for reproducible and shareable neuroscience research
Source: eLife. 2026 Nov 3;14:RP108089. doi: 10.7554/eLife.108089 (PMC13730526; doi:10.7554/eLife.108089)
Supplement: Peer review history

# Editor report: eLife Assessment

**DOI:** 10.7554/eLife.108089.3.sa0

**Authors:** Adrien Peyrache

**Affiliations:** McGill University Canada

**Keywords:** Convincing, Valuable

This **valuable** study presents a framework for a shareable data analysis pipeline aimed at improving reproducibility in neuroscience. The evidence for robustness and inter-laboratory operability is **convincing**. Overall, this work will be of interest to neuroscientists engaged in the analysis of large-scale neuronal recordings.

# Referee report: Reviewer #1 (Public review)

**DOI:** 10.7554/eLife.108089.3.sa1

Summary

The manuscript by K.H. Lee et al. presents Spyglass, a new open-source framework for building reproducible pipelines in systems neuroscience. The framework integrates the NWB (Neurodata Without Borders) data standard with the DataJoint relational database system to organize and manage analysis workflows. It enables the construction of complete pipelines, from raw data acquisition to final figures. The authors demonstrate their capabilities through examples, including spike sorting, LFP filtering, and sharp-wave ripple (SWR) detection. Additionally, the framework supports interactive visualizations via integration with Figurl, a platform for sharing neuroscience figures online.

Strengths:

Reproducibility in data analysis remains a significant challenge within the neuroscience community, posing a barrier to scientific progress. While many journals now require authors to share their data and code upon publication, this alone does not ensure that the code will execute properly or reproduce the original results. Recognizing this gap, the authors aim to address the community's need for a robust tool to build reproducible pipelines in systems neuroscience.

Comments on revisions:

In this revised version, the authors have addressed the majority of the concerns raised in the initial review. The manuscript is clearer, the documentation and explanations have been strengthened, and several important practical issues-particularly regarding usability, terminology, and deployment-have been meaningfully improved. While the framework continues to position itself both as a flexible analysis environment and as a mechanism for freezing and preserving reproducible pipelines, the authors have clarified their rationale for maintaining this dual role. I have no additional comments at this stage.

# Referee report: Reviewer #2 (Public review)

**DOI:** 10.7554/eLife.108089.3.sa2

Summary:

Lee et al. introduce Spyglass, an open-source Python framework designed to tackle the reproducibility crisis in systems neuroscience by integrating the Neurodata Without Borders (NWB) standard with DataJoint relational databases. The framework aims to standardize data ingestion, preprocessing, analysis pipelines, and data sharing for complex electrophysiological and behavioral experiments.

Strengths:

(1) Handling of Complex Workflows: The architectural design is pragmatic and robust. Features such as the "cyclic iteration" motif for spike-sorting curation and the "merge" motif for consolidating multiple data streams effectively handle the iterative nature of data processing without incurring database bloat.

(2) Ecosystem Integration: The revised manuscript clarifies that Spyglass acts as a community hub, explicitly detailing its integration with established tools like SpikeInterface, DeepLabCut, GhostiPy, MoSeq, and Pynapple.

(3) Pipeline Clarity & Practical Demonstration: The addition of Supplementary Figure 1, in conjunction with Figure 5, successfully maps out the complex, multi-step decoding workflow for both the UCSF and NYU datasets. Together, these figures tell a complete and compelling story of how this pipeline can be used in practice, providing much-needed visual clarity on how raw data moves through the database to generate final results.

Appraisal:

The authors have successfully achieved their aims. Spyglass is a highly functional system capable of handling the heavy lifting of data management. The revisions have significantly improved transparency regarding the tool's limitations and its onboarding process, making it a highly attractive blueprint for labs aiming to adhere to FAIR principles.

# Author comment: Author response

**DOI:** 10.7554/eLife.108089.3.sa3

**Authors:** Kyu Hyun Lee, Eric L Denovellis, Ryan Ly, Jeremy Magland, Jeff Soules, Alison E Comrie, Daniel P Gramling, Jennifer A Guidera, Rhino Nevers, Philip Adenekan, Chris Brozdowski, Samuel R Bray, Emily Monroe, Ji Hyun Bak, Michael E Coulter, Xulu Sun, Emrey Broyles, Donghoon Shin, Sharon Chiang, Cristofer Holobetz, Andrew Tritt, Oliver Rübel, Thinh Nguyen, Dimitri Yatsenko, Joshua Chu, Caleb Kemere, Samuel Garcia, Alessio Paolo Buccino, Loren Frank

**Affiliations:** University of California, San Francisco San Francisco United States; University of California, San Francisco San Francisco United States; Lawrence Berkeley National Laboratory Berkeley United States; Flatiron Institute New York United States; Center for Computational Mathematics, Flatiron Institute New York United States; Department of Physiology, University of California, San Francisco; Kavli Institute for Fundamental Neuroscience, University of California, San Francisco San Francisco United States; Graudate Program in Neural and Behavioral Sciences, University of Tubingen Tubingen Germany; Department of Physiology, University of California, San Francisco; Kavli Institute for Fundamental Neuroscience, University of California, San Francisco; UCSF-UC Berkeley Graduate Program in Bioengineering, University of California, San Francisco San Francisco United States; University of California San Francisco San Francisco United States; Department of Physiology, University of California, San Francisco; Kavli Institute for Fundamental Neuroscience, University of California, San Francisco San Francisco United States; Department of Physiology, University of California, San Francisco; Kavli Institute for Fundamental Neuroscience, University of California, San Francisco San Francisco United States; Department of Physiology, University of California, San Francisco; Kavli Institute for Fundamental Neuroscience, University of California, San Francisco San Francisco United States; Department of Physiology, University of California, San Francisco San Francisco United States; Department of Physiology, University of California, San Francisco San Francisco United States; University of California, San Francisco San Francisco United States; UCSF San Francisco United States; Department of Physiology, University of California, San Francisco; Kavli Institute for Fundamental Neuroscience, University of California, San Francisco San Francisco United States; Department of Physiology, University of California, San Francisco; Kavli Institute for Fundamental Neuroscience, University of California, San Francisco; UCSF-UC Berkeley Graduate Program in Bioengineering, University of California, San Francisco San Francisco United States; Department of Neurology, University of California, San Francisco San Francisco United States; Sainsbury Wellcome Centre, University College London London United Kingdom; Lawrence Berkeley National Laboratory Berkeley United States; Lawrence Berkeley National Laboratory Berkeley United States; DataJoint Houston United States; DataJoint Houston United States; Department of Electrical and Computer Engineering, Rice University Houston United States; Rice University Houston United States; Centre National de la Recherche Scientifique Lyon France; Allen Institute for Neural Dynamics Seattle United States; University of California,San Francisco, Howard Hughes Medical Institute San Francisco United States

The following is the authors’ response to the original reviews.

**Reviewer #1 (Public review):**

Summary

The manuscript by K.H. Lee et al. presents Spyglass, a new open-source framework for building reproducible pipelines in systems neuroscience. The framework integrates the NWB (Neurodata Without Borders) data standard with the DataJoint relational database system to organize and manage analysis workflows. It enables the construction of complete pipelines, from raw data acquisition to final figures. The authors demonstrate their capabilities through examples, including spike sorting, LFP filtering, and sharpwave ripple (SWR) detection. Additionally, the framework supports interactive visualizations via integration with Figurl, a platform for sharing neuroscience figures online.

Strengths:

Reproducibility in data analysis remains a significant challenge within the neuroscience community, posing a barrier to scientific progress. While many journals now require authors to share their data and code upon publication, this alone does not ensure that the code will execute properly or reproduce the original results. Recognizing this gap, the authors aim to address the community's need for a robust tool to build reproducible pipelines in systems neuroscience.

We appreciate the summary and the recognition of the key need for maximally reproducible scientific workflows.

Weaknesses:

The issues identified here may serve as a foundation for future development efforts.

(1) User-friendliness:

The primary concern is usability. The manuscript does not clearly define the intended user base within a modern systems neuroscience lab. Improving user experience and lowering the barrier to entry would significantly enhance the framework's potential for broad adoption. The authors provide an online example notebook and a local setup notebook. However, the local setup process is overly complex, with many restrictive steps that could discourage new users. A more streamlined and clearly documented onboarding process is essential. Additionally, the lack of Windows support represents a practical limitation, particularly if the goal is widespread adoption across diverse research environments.

We agree that usability is critical, and we now clarify that Spyglass

“… is designed to be used by everyone in a laboratory who works with the data, both as a general-purpose tool to enable the development of new analysis pipelines and a tool that allows those pipelines and associated results to be frozen and packaged to enable reproducibility…”

To address the local setup issue, we have now created an interactive quick start program to guide new users through the setup (scripts/install.py). It now leads the user through a few prompts with sensible defaults to reduce the complexity of the setup. It aids the user in installing the Spyglass dependencies and creating the Data joint configuration file. We also validate the configuration to make sure the set up was successful (scripts/validate.py). Combined, these should reduce the complexity and set up time for most users while allowing expert users to configure Spyglass as they need. We thank the reviewer for the suggestion.

We also agree that the lack of support for Windows is a key issue, and that is something we plan to address in the coming years. We note that it may be possible to run Spyglass under the Windows Subsystem for Linux (WSL 2), which allows users to run Linux programs on a Windows machine without the need for a virtual machine or dual boot setup.

(2) Dependency management and long-term sustainability:

The framework depends on numerous external libraries and tools for data processing. This raises concerns about long-term maintainability, especially given the short lifespan of many academic software projects and the instability often associated with Python's backward compatibility. It would be helpful for the authors to clarify how flexible and modular the pipeline is, and whether it can remain functional if upstream dependencies become deprecated or change substantially.

This is a very good point that reflects a broad challenge to maintainability and reproducibility. We now explicitly raise this point in our Limitations section, and note that

“…even in cases where reproducing a result would require installing older versions of software, the results themselves remain accessible within NWB files referenced in Spyglass, ensuring that previous results can be built on even as packages evolve.”

The merge table pattern also allows us to update (version) our pipelines as software changes. For example, we have already done so for changes in SpikeInterface versions for the version 1 pipeline for spike sorting. New and older versions of the pipeline (v0 and v1) are accessed through the merge table SpikeSortingOutput. This allows the user to have consistent results despite the version change.

(3) Extensibility for custom pipelines:

A further limitation is the insufficient documentation regarding the creation of custom pipelines. It is unclear how a user could adapt Spyglass to implement their own analysis workflows, especially if these differ from the provided examples (e.g., spike sorting, LFP analysis that are very specific to the hippocampal field). A clearer explanation or example of how to extend the framework for unrelated or novel analyses would greatly improve its utility and encourage community contributions.

Here we failed to provide the required links to the documentation. We now explicitly refer to documentation on Custom Pipeline, which include a link to a YouTube video walking users through the creation of such a pipeline:

Specifically, Spyglass uses DataJoint syntax to define tables as Python classes (see online documentation on Custom Pipelines and this video for examples).

(4) Flexibility vs. Standardization:

The authors may benefit from more explicitly defining the intended role of the framework: is Spyglass designed as a flexible, general-purpose tool for developing custom data analysis pipelines, or is its primary goal to provide a standardized framework for freezing and preserving pipelines post-publication to ensure reproducibility? While both goals are valuable, attempting to fully support both may introduce unnecessary complexity and result in a tool that is not well-suited for either purpose. The manuscript briefly touches on this tradeoff in the introduction, and the latter-pipeline preservation-may be the more natural fit for the package. If so, this intended use should be clearly communicated in the documentation to help users understand its scope and strengths.

We appreciate this point, and have now clarified in the beginning of the Results section that

It is both a general-purpose tool to enable the development of new analysis pipelines and a tool that allows those pipelines and associated results to be frozen and packaged to enable reproducibility.

In practice, our lab uses Spyglass to systematize analyses to enable rapid application across many datasets. Then, once a paper has been finalized, we can export the data and the code in a package that enables reproduction. Being able to do both things is, in our view, a key strength of Spyglass. More broadly, we feel it is critical that there be a clear path for users to take their analysis code and make it reproducible. That process normally involves a very substantial amount of work, and our goal was to reduce the burden on users and make this a straightforward extension of how analyses are carried out.

Impact:

This work represents a significant milestone in advancing reproducible data analysis pipelines in neuroscience. Beyond reproducibility, the integration of cloud-based execution and shareable, interactive figures has the potential to transform how scientific collaboration and data dissemination are conducted. The authors are at the forefront of this shift, contributing valuable tools that push the field toward more transparent and accessible research practices.

We appreciate this positive assessment.

**Reviewer #1 (Recommendations for the authors):**

(1) "The authors write: ‘the relational database, a well-known data structure that uses tables to organize data.’ This phrasing may be misleading… It would be more accurate to describe them as ‘well-established’ rather than ‘well-known.’"

We have made this change.

(2) The statement "It makes it easy to apply the same analysis to multiple datasets, as users need to specify only the data and parameters for computation ("what") rather than the execution details ("how")." would benefit from further elaboration. Specifically, how does this approach compare in practice to using a simple configuration file (e.g., YAML or JSON) to manage parameters and execution logic? A comparison or example would help ground the claim.?"

We agree one could in principle do something similar with configuration files, but this is a discipline that the user must impose on themselves, as configuration files in general have no constraint on how they are to be used. On the other hand, a system like Spyglass enforces the separation of data from parameters by design. We have now added a brief comment on this point in the Results:

“It provides a structure to organize and systematize the analysis parameters, data, and outputs into different tables. This contrasts with user-generated configuration files where each user could adopt their own idiosyncratic approach to specifying parameters and data.”

We also come back to this point in the Discussion:

Other approaches do away with the relational database altogether. For example, DataLad uses version control tools such as git and git-annex to manage both code and data as files [39]. This enables the creation of a data analysis environment and decentralized data sharing. For building analysis pipelines, it may be combined with other tools for managing the sequential execution of scripts. For example, Snakemakeb[40] (and related projects such as Cobrawap [41]) allows the users to gather and define the input, output, and the associated scripts to execute for each analysis step, thereby tracking the dependency between steps. But because these tools do not provide any formal structure for data analysis or parameter specification, they lack the advantages of the relational database that we discussed, such as being able to easily organize or search for the records of previous analysis based on specific parameters, efficient data sharing and access management to multiple users, and built-in data integrity checks based on constraints native to the database (e.g. primary keys).

(3) The sentence ‘It enables easy access to multiple datasets via queries’ may overstate the benefit… clarify what specific advantages database queries offer.

We agree that this is an important feature and we added the following as an example of the advantage of being able to query the database:

It enables easy access to multiple datasets via queries (e.g. to find all datasets with recordings from a particular brain region or that used a particular behavioral paradigm)

(4) Specifically, Spyglass uses DataJoint syntax to define tables as Python classes’ lacks clarity… Expanding this explanation with a brief, concrete example would

We agree that this sentence does not provide information on how to use DataJoint syntax to define a table. We carefully considered adding that syntax to the manuscript, but we are concerned that doing so here and in other places where syntax examples could be used would decrease the readability of the document. We also noted that other papers that present analysis frameworks typically provide much less information.

Nevertheless, it is clear that users would benefit from a concrete example, and as we mentioned above, we have added a link to the documentation describing how to make custom schema and pipelines, as well as a YouTube video that we created to walk users through this process.

(5) The authors write: "Selection tables associate parameter entries with data object entries." This terminology is confusing. From a naming perspective, it is not immediately obvious what a "selection table" is or how it differs from other components. Moreover, shouldn't parameter entries be associated with a specific pipeline rather than directly with data objects? Further clarification is needed. "

We appreciate that our terminology was not clear. The idea behind a selection table is that there are many data entries and many potential sets of parameters that can be used to analyze each of those entries. We have now revised this section of the text and added an explanatory paragraph:

An analysis pipeline consists of sets of tables downstream of the Common tables. In each step in the analysis, the user populates one of four table types (Figure 2A):

Data tables contain pointers to data objects in either the original NWB file or ones generated by an upstream analysis.

Parameter tables contain a list of the parameters needed to fully specify the desired analysis.

Selection tables allow users to select and pair a data entry and a parameter entry, defining the input to the Compute table.

Compute tables execute the computations to carry out the analysis using the Data and Parameters specified in the Selection table entry. These results are then stored and can serve as Data for downstream analysis.

This design has multiple features that we have found to be beneficial. First, Parameter tables store the full set of parameters needed to specify a given analysis. For example, a Parameter table entry for a firing rate analysis of a single neuron might specify the bin size and smoothing to be used for that analysis. Multiple such entries can be defined, allowing a user to select the most appropriate one for the question being addressed. Second, because Selection tables specify which Parameter table entry was used for a given analysis on the associated Data table entry, they provide the key information needed to know which parameters were used to generate the entry in the downstream Compute table. Third, it is simple to associate a given Data table entry with multiple Parameter table entries and then re-run the analysis on those pairs. This enables a user to understand how their choice of parameters impacts their results, something that is otherwise difficult to manage and track.

(6) Including ‘fitting state-space models’ as a standard example may be misleading… Presenting it as a routine task might set unrealistic expectations."

We agree and have changed “standard” to “a diverse range of”.

(7) Figure 2 would benefit from clearer sequential logic. For example, the object ‘LFPSelection’ appears after a method call referencing it."

We agree that the figure was not explained adequately. We now make it clear in the caption that the method call creates the entry in the LFPSelection table, and is thus upstream of the picture of the table entry that was created.

(8) Example 3 would be strengthened by a comparison to SpikeInterface, a framework increasingly adopted by the community."

Here we clearly did not explain the spike sorting pipeline sufficiently thoroughly. As we now clarify in the text:

This pipeline uses SpikeInterface [19] to perform the operations critical for spike sorting, but also tracks all of the parameters used and provides a system for tracking multiple sorting curations.

Thus, Spyglass takes advantage of the special purpose routines within SpikeInterface, but also provides an organizational framework for the outputs, and, equally critically, allows direct use of the outputs of sorting in downstream analyses with the ability to go back and know which sorting parameters were used for that analysis.

(9) The authors state: "These are saved as Docker containers and optionally uploaded to DANDI." However, it is unclear how end users are expected to interact with these containers. Additional guidance or an example interaction would be valuable.

We agree that this interaction was not described in the text, and we have now added the following to explain how a user might interact with these containers:

...This can be done by (i) hosting the database on the cloud and granting access to users outside the lab; or (ii) exporting and sharing parts of the database that were used by the project. Spyglass facilitates the second option by providing functions that automatically log the table entries and NWB files used for creating figures of a manuscript in a Python environment (Table 1, 05_Export). The dependencies of these entries are traced through the database to compile the complete set of raw, intermediate, and plotted NWB files and their corresponding database entries. These are stored in the `Export` table, which also generates a bash script to create SQL dumps of the identified database entries.

To upload these files to DANDI, users must first register a new dandiset for their project and record their API and dandiset ID. With this information, they can then use the method `DandiPath.compile_dandiset()` to automatically validate, organize, and upload all project files to the DANDI archive. Additionally, this process stores the archive information for each file in the `DandiPath` table, allowing `fetch_nwb` to automatically stream data from the DANDI cloud storage when not available locally.

To create a sharable Docker image of the project, we provide a template repository spyglass-export-docker. Users first download a local copy of this repo and copy the SQL dump file, environment YAML, and figure-generating notebooks generated during spyglass export into the appropriate folders. Running the provided Docker compose scripts then generates two linked Docker containers: one running the reconstructed spyglass SQL database, and a second connected to this database and running a JupyterHub with a Python environment matching that used when generating the figures. These can be readily shared with new users to provide them immediate access to all steps of the analysis process and the corresponding data through DANDI streaming

(10) The phrase "not requiring a central location to track available files and providing a user-friendly Python API" is somewhat vague. Does this imply that multiple sources can exist for the same NWB file? How does the system handle potential version conflicts, such as when an NWB file is modified locally? A clearer explanation would help users understand the system's behavior in collaborative scenarios. "

This is an important point that we now explain in the manuscript:

Critically, the downloaded files are never modified locally within Spyglass and attempt to access a modified file would result in a DataJoint error. This ensures that each user is working on the same underlying data even if they are at different sites.

To provide interested readers with more details, we also now point them to the repo for more information:

We point interested readers to the Kachery GitHub repo (https://github.com/magland/kachery) for further descriptions.

(11) "The concept of a ‘kachery zone’ in Figure 4 is ambiguous. Is this storage local or in the cloud? If a third-party storage system is involved, it should be explicitly labeled and described in the diagram."

We agree that the depiction of a Kachery zone in Figure 4 is hard to understand. For the reviewer’s reference, a Kachery zone defines a list of users that have permissions to upload and download a particular set of files that have been linked to that zone. This is a explained in the tutorials, and to simplify the figure we have replaced the Kachery zone with a remote computer.

(12) If one of the manuscript's goals is to showcase the functionality of the pipeline, Figure 5 would be more informative if it also illustrated the workflow or steps involved in generating the displayed figures.

We have added a supplementary figure (Supplementary Figure 1) related to figure 5 that illustrates the main data workflow used in generating the figure. In addition, we note that the code for generating the figure 5 and supplemental are included in the code repository for the paper (https://github.com/LorenFrankLab/spyglass-paper/).

(13) In the conclusion, the authors write: "By contrast, Spyglass begins with a shared data format that includes the raw data and offers both transparent data management and reproducible analysis pipelines using a formal data structure." However, the tools discussed in the previous paragraph seem to offer similar capabilities. The real challenge in transparent data management often lies in the technical overhead associated with setting up and maintaining a database, particularly when collaborating across labs.

Here we may not have explained the differences between Spyglass and these other approaches sufficiently clearly. The various tools mentioned in the paragraph above this one do not begin with a shared format nor do they include a formal data structure. That said, we agree that maintaining a database accessible across labs is a key challenge. We note here that we provide tutorials to ease this process, which are linked and described in the manuscript (e.g. Table 1).

(14) Specifying a preferred IDE… may not be necessary. This recommendation could be made optional or omitted."

We agree that it may not be necessary, but we have also noted that users come to Spyglass with a very wide range of expertise, and in our lab it has been helpful to specify the IDE.

**Reviewer #2 (Public review):**

Summary:

This valuable paper presents Spyglass, a comprehensive software framework designed to address the critical challenges of reproducibility and data sharing in neuroscience.

The authors have developed a robust ecosystem built on community standards such as NWB and DataJoint, and demonstrate its utility by applying it to datasets from two independent labs, successfully validating the framework's ability to reproduce and extend published findings. While the framework offers a powerful blueprint for modern, reproducible research, its immediate broad impact may be tempered by the significant upfront investment required for adoption and its current focus on electrophysiological data. Nevertheless, Spyglass stands as an important and practical contribution, providing a well-documented and thoughtfully designed path toward more transparent and collaborative science.

Strengths:

(1) Principled solution to a foundational challenge:

The work offers a concrete and comprehensive framework for reproducibility in neuroscience, moving beyond abstract principles to provide an implemented, end-to-end ecosystem.

(2) Pragmatic and robust architectural design:

Features such as the "cyclic iteration" motif for spike-sorting curation and the "merge" motif for pipeline consolidation demonstrate deep, practical experience with neurophysiological analysis and address real-world challenges.

(3) Cross-laboratory validation:

The successful replication and extension of published hippocampal decoding findings across independent datasets strongly support the framework's utility and underscore its potential for enabling reproducible science.

(4) Accessibility through documentation and demos:

Extensive tutorials and the availability of a public demo environment lower some of the barriers to adoption.

We appreciate the Reviewer’s recognition of these strengths.

Weaknesses:

(1) High barrier to adoption:

The requirement to convert all data into NWB, maintain a relational database, and train users in structured workflows is a significant hurdle, particularly for smaller labs.

We agree that this is a significant hurdle, but we also believe that it comes with many advantages. It is also increasingly easy to do given the many community-supported tools, regardless of how much resource the lab has. These points are discussed in detail in “Why NWB?” section.

We also note that, to our knowledge, there is no simpler alternative that provides the key features of Spyglass.

(2) Limited tool integration:

The current pipelines, while useful, still resemble proof-of-principle demonstrations.

Closer integration with established analysis libraries such as Pynapple and others could broaden the toolkit and reduce duplication of effort.

Here we clearly failed to explain that we have integrated other libraries, including Pynapple. We now make this clear in the Results section:

Our goal was take advantage of other open source packages, and we have therefore integrated support for Pynapple [21], a general purpose neural data analysis package. We also built our pipelines to take advantage of other community-developed, open-source packages, like GhostiPy [20], SpikeInterface [19], DeepLabCut [2] and MoSeq [29].

We also have added a specific reference to the relevant function call in the Practical use cases and extensions section:

For example, the user can conveniently read specific data types from the NWB file by first ingesting it into Spyglass and accessing database tables with Spyglass functions (e.g. fetch_nwb) or even load those objects in a format compatible with Pynapple [21] (fetch_pynapple).

Pynapple support is actually aided by our design choice of relying on NWB. Because NWB files can be loaded by Pynapple, any analysis that uses a NWB file that can be read by Pynapple can be loaded as a Pynapple object. We have provided methods to do so.

(3) Experimental metadata support:

While NWB provides a solid foundation for storing neurophysiology data streams, it still lacks broad and standardized support for experimental metadata, including descriptions of conditions, subject details, and procedures, as well as links across datasets. This limitation constrains one of Spyglass's key promises: enabling reproducible, crosslaboratory science. The authors should clarify how Spyglass plans to address or mitigate this gap - for example, by adopting or contributing to metadata extensions, providing templates for experimental conditions, or integrating with complementary systems that manage metadata across datasets.

This is an important point. First, NWB provides methods for creating new metadata extensions, and our laboratory has contributed to multiple such extensions and have adopted metadata extensions as they come to exist (for example, we are currently integrating the ndx-pose extension, which has broader support for pose estimation algorithms such as DLC and SLEAP, enabling us to capture relationships between body parts). These extensions, once incorporated into NWB, make it easy to create parallel Spyglass tables that read in the associated metadata. Second, we note that by storing the metadata from the NWB file in a database, Spyglass naturally supports searches across datasets where the metadata is the same (e.g. all the datasets from a given subject or using a given behavioral apparatus).

That said, for these searches to be easy, the underlying NWB files need to use the same ontologies (naming systems). Creating shared naming systems within and across labs is very challenging, but even here having a database helps greatly, as it provides a way to find all the names used for a given field and to thereby make an effort to standardize them.

Finally, while Spyglass aims to enable reproducibility, it will not be possible to solve all standardization issues of the field. We believe that Spyglass is an important step forward in standardization and reproducibility in that it encourages users to use the same data format and processing. To our knowledge, there is no software like it in the field of systems neuroscience. Limitations of the field and of current progress does not invalidate the contribution of Spyglass as a framework.

We now mention all these issues in the Limitations section of the Discussion.

(4) Cross-laboratory interoperability:

While demonstrated across two datasets, the manuscript does not fully address how Spyglass will handle the diversity of metadata standards, acquisition systems, and labspecific practices that remain major obstacles to reproducibility.

We agree that the current version of Spyglass does not fully address this diversity. Neverless, we note that the NWB standard is increasingly widely adopted in our field, and that by building on this standard, it is much similar to create structures that store relevant data across labs.

(5) Visualization limitations:

Beyond the export system and Figurl, NWB offers relatively few options for interactive data exploration. The ability to explore data flexibly and discover new phenomena remains limited, which constrains one of the potential strengths of standardized pipelines.

We agree that there are many other tools, and we have considered additional integrations. We have chosen not to proceed in this direction because the various visualization tools are well constructed, and therefore already easy to use with data retrieved from Spyglass. Thus, users can choose to use Matplotlib, Seaborn, or any of many other visualization tools and apply thos to data accessed through Spyglass without the need for more explicit integration.

Spyglass is well-positioned to become a community framework for reproducible neuroscience workflows, with the potential to set new standards for transparency and data sharing. With expanded modality coverage, tighter integration of existing community tools, stronger solutions for cross-lab interoperability, and richer visualization capabilities, it could have a transformative impact on the field.

We appreciate this summary and will continue to try to make Spyglass more powerful, generalizable, and accessible to the community.

**Reviewer #2 (Recommendations for the authors):**

(1) Documentation/User onboarding:

While extensive documentation exists, new users may feel overwhelmed. A single Quickstart or "golden path" guide and a one-command validation script would substantially improve usability.

As mentioned in the response to reviewer 1, we have added an interactive quickstart program to walk users through installation and setup (scripts/install.py) and validate the install (scripts/validate.py). This should greatly reduce the complexity of the set-up process and allow new users to use Spyglass quickly and confidently. We thank the reviewer for the suggestion.

(2) Permission handling and multi-user scaling:

Current ad hoc solutions (like cautious deletes) may not scale well in large collaborations. This should be acknowledged, but it is not a fatal weakness given the framework's early stage.

This is a fair point and we now mention this when cautious delete is introduced in the Methods:

Though this is not a formal permission-management system, it serves to prevent accidental deletions. We note that this system does incur additional overhead, and while that has not been an issue for us, it is possible that this would become problematic in use for much larger cross-laboratory collaborations.

(3) Benchmarking and performance evaluation:

"More systematic testing (e.g., reproducibility across independent users, computational efficiency) would be reassuring, but the lack of it does not invalidate the proof-of-principle demonstration. "

We agree. So far at least two other labs have adopted this system and we are working with a consortium funded by the Simons Foundation to use Spyglass as a data sharing system across a larger number of labs.

(4) Support for Cloud solution:

To lower the barrier to adoption, the authors should consider cloud integration, such as preconfigured Docker/Cloud templates or hosted options, so end-users do not need to maintain databases and storage locally.

We agree that cloud-based solutions could be a good option for some labs, although we note that the cost of cloud-based computing can be very high. There is also the burden of moving and storing the data to where it needs to be processed, which can be particularly time intensive with the large-scale data being generated by many laboratories.

At the reviewer’s suggestion, we have added a docker-compose support to lower the barrier to adoption. This includes:

docker-compose.yml with health checks and persistent storage

.env.example configuration template

This allows one-command database setup: `docker compose up –d`

(5) Integration of greater modalities:

The authors should consider expanding support to other major data types, particularly calcium imaging, photometry, and other optical physiology data.

We entirely agree that pipelines to ingest and process these data types would be very valuable, and we would welcome collaborations with experts and the general community to build these pipelines. We are, for example, working with a collaborating lab on a photometry pipeline. However, we only have so many people to build and maintain Spyglass, so we are limited by the capacity and expertise of our developers.

(6) Integrate more community tools:

Closer integration with community tools such as Pynapple, Neurosift, and SpikeInterface would broaden functionality and position Spyglass as a hub rather than a parallel ecosystem.

As we mentioned in our responses to Reviewer 1, we entirely agree, and in fact we have already integrated Pynapple support into Spyglass. Because we store files in the NWB format and Pynapple supports NWB, it was easy for us to convert any data we have into the Pynapple format upon request, thus making it easily analyzable by the Pynapple package. Moreover, we use SpikeInterface for the SpikeSorting pipline, and similarly provide pipelines built on other open source projects. As we now clarify in the text:

Spyglass includes pipelines for a diverse range of analysis tasks in systems neuroscience, such as the analysis of LFP, spike sorting, video and position processing, and fitting state-space models for decoding neural data. Tutorials for all pipelines are available on the Spyglass documentation website (Table 1). Our goal was take advantage of other open source packages, and we have therefore integrated support for Pynapple [21], a general purpose neural data analysis package. We also built our pipelines to take advantage of other community-developed, open-source packages, like GhostiPy [20], SpikeInterface [19], DeepLabCut [2] and MoSeq [29].

(7) Direct Dandi archive upload functionality:

Scripts and tutorials for uploading data directly from Spyglass to DANDI, with validation of metadata completeness, would provide users with a direct pipeline from raw data to a public archive.

The tutorials for DANDI upload are included as part of the export tutorial notebook (https://lorenfranklab.github.io/spyglass/latest/notebooks/05_Export/). We agree that this was not apparent from the manuscript before and have noted this within the Manuscript table describing these notebooks.
